# Revision and complication rates in adult shunt surgery: a single-institution study

**DOI:** 10.1007/s00701-020-04526-z

**Published:** 2020-10-31

**Authors:** Nadia Mansoor, Ole Solheim, Oddrun A. Fredriksli, Sasha Gulati

**Affiliations:** 1 Department of Neurosurgery, St. Olavs University Hospital, Trondheim, Norway; 2 Nevroklinikken, St. Olavs Hospital, 7006 Trondheim, Norway; 3 Department of Neuromedicine and Movement Science, Norwegian University of Science and Technology (NTNU), Trondheim, Norway

**Keywords:** Ventriculoperitoneal shunt, Shunt surgery, Shunt revision, Shunt complications

## Abstract

**Background:**

CSF diversion with shunt placement is frequently associated with need for later revisions as well as surgical complications. We sought to review revision and complication rates following ventriculoperitoneal, ventriculoatrial and cystoperitoneal shunt placement in adult patients, and to identify potential risk factors for revision surgery and postoperative complications.

**Method:**

Included patients were adults (≥ 18 years) who underwent primary shunt insertion at St. Olavs Hospital in Trondheim, Norway, from 2008 through 2017. The electronic medical records and diagnostic imaging from all hospitals in our catchment area were retrospectively reviewed. Follow-up ranged from 1 to 11 years. Complications were graded according to the Landriel Ibañez classification system.

**Results:**

Of the 227 patients included, 47 patients (20.7%) required revision surgery during the follow-up. In total, 90 revision surgeries were performed during follow-up. The most common cause for the *first* revision was infection (5.7%) and for *all* revisions proximal occlusion (30.0%). A total of 103 patients (45.4%) experienced ≥ 1 complication(s). Mild to moderate complications (grade I and II) were detected in 35.0% of all procedures. Severe or fatal complications (grade III and IV) were observed in 8.2% of all procedures*.* Urinary tract infections and pneumonia were common postoperatively (13.9% and 7.3%, respectively), and the most common IIb complication was shunt misplacement (proximally or distally). Two out of fourteen deaths within 30 days were directly associated with surgery. We did not find that aetiology/indication, age or gender influenced the occurrence of revision surgery or a grade III or IV complication.

**Conclusions:**

Shunt surgery continues to be a challenge both in terms of revision rates and procedure-related complications. However, the prediction of patients at risk remains difficult. A multidimensional focus is probably needed to reduce risks.

## Background

Shunt surgery is frequently performed in most neurosurgical departments and remains the most common permanent cerebrospinal fluid (CSF) diversion method in patients with hydrocephalus (HC). Shunt surgery accounted for almost 5% of all surgeries in our department (primary shunt insertion and shunt revisions of any kind) in 2019.

Unfortunately, CSF diversion with shunt placement is still associated with a significant risk of short- and long-term complications, and the patient burden associated with shunt and revision surgery should not be underestimated. The cost and strain of shunt surgery (primary insertion and complications leading to revision surgery) on the health care system have been estimated to total around one billion dollars in the USA [14]. Studies have indicated that factors such as age, aetiology and/or type of hydrocephalus (communicating, non-communicating) may be independent risk factors for shunt complications, revision rates and overall survival [1, 12, 13, 17–19, 23], whilst others found no such association [4].

A recent Danish study compared two cohorts of patients approximately 50 years apart and found no improvement in revision rates [9]. This indicates that despite more than 50 years of experience with advancements in shunt technology and perioperative care, revision rates remain unfortunately unchanged. Lack of progress in reducing revision rates has also been reported by others [21].

The rate of revisions cited in previous studies is generally reported to be around 20–30% in the adult populations [12, 19, 23], but higher rates have also been reported, especially in paediatric populations [9]. There have been many attempts to prevent and reduce complication rates, and a recent randomised trial found that the use of antibiotic-impregnated intraventricular catheters reduced infection rates from 6 to 2%, although the total revision rate was similar in the two groups [10]. The use of antibiotic catheters has also been reported to reduce costs in a paediatric population due to shorter hospital stays, decreased inpatient complication rates and less frequent use of multiple antibiotic regimes [20]. Still, few shunt studies address *overall* complication rates and tend to focus on revision rates only.

In the current study, we sought to review both revision rates *and* 30-day complication rates following shunt surgery in adults over a 10-year period and to explore possible predictors for the need for shunt revision and perioperative complications.

## Methods

Ethical approval and waiver of the requirement for obtaining patient consent were granted by the Regional Committee for Medical Research (REK 2017/1796).

### Data collection

We retrospectively reviewed all adults aged 18 years and older, who underwent primary shunt insertion at St. Olavs University Hospital, Trondheim, Norway, from January 2008 through December 2017. All patients were followed for at least 1 year (through 31st of December 2018), range 1–11 years. Electronic medical records from all seven hospitals in our geographical catchment area were reviewed.

We used the Nomesco Classification of Surgical Procedures to retrieve records of all patients who had undergone any form of CSF diversion surgery (AAF 00, AAF 05, AAF 15, AAF 20, AAF 25, AAF 40, AAF99, JAL 50, JAL 51) in the given time period. Patients were then reviewed individually, and only patients who underwent primary ventriculoperitoneal, ventriculoatrial, or cystoperitoneal shunt operations in the given time period were included. Patients who underwent revision surgery in the given time period, but with primary shunt insertion outside the given time period, were excluded.

We extracted baseline data for all patients including gender, age at shunt surgery, indication, comorbidities, ASA risk group and status at follow-up (alive or dead). We also extracted data on the surgical procedure itself; surgery duration, type of shunt and valve inserted, whether antibiotic prophylaxis was given, whether the patient had previous external ventricular drain prior to the shunt procedure, and the postoperative Kakarla score [8]. We extracted data on all shunt revisions performed on these patients throughout the follow-up period, as well as complications occurring within 30 days of shunt surgery. Shunt infection was defined by the finding of significantly increased leukocytes in CSF specimen with or without evidence of microbes (microscopy), together with lab findings (elevated leukocytes and C-reactive protein in serum) and clinical finding (fever, headache, reduced GCS) in awake patients. If any of these were present and resulted in *either* complete removal of shunt or temporary externalisation of the shunt together with intravenous use of antibiotics, this was registered as a shunt infection. The occurrence of superficial wound infection requiring oral antibiotics only, and where no surgical measures were taken, was not registered as shunt/CNS infection. Complications were classified according to the classification of neurosurgical complications defined by Landriel Ibañez et al. [6]. If patients had more than one complication, the complication with the highest grade was noted.

### Data analysis

Statistical analyses were performed using SPSS version 25 (IBM corporation). The *p* value for significance was set to 0.05. Calculation of frequencies was performed for all categorical data. To establish whether there were any differences between the patients who underwent revision(s) and the non-revision group, we used the chi-square or Fischer exact test for all categorical data and the independent *t* test for all continuous data. Normality for continuous data was tested using the Sapiro-Wilk test and visualised using histograms and Q-Q plots. The Mann-Whitney *U* test was used to compare the two groups if continuous variables were skewed. A cox regression model was developed in search of risk factors associated with the need for revision surgery, and a binary regression model was developed in the search for risk factors of occurrence of revision surgery or severe or fatal postoperative complications (Landriel Ibañez grade III or IV). Revision-free shunt survival in the revision group was estimated from a Kaplan-Meier (KM) analysis.

## Results

### Patient demographics

Patient demographics are presented in Table 1. We included 227 patients, with a mean age of 58.4 years ± 15.6 (range 18–89). The most common aetiology for primary shunt surgery was hydrocephalus following subarachnoid haemorrhage (35.7%), followed by normal pressure hydrocephalus (NPH) (24.2%), and patients with tumour and/or malignancy (18.9%). Severe comorbidity, defined as ASA > 2 at the time of index surgery, was present in 50.3% of the patients. Patients who underwent shunt revisions were younger (mean age 53.0 vs. 59.8 years, *p* = 0.014) and fewer were deceased at the last follow-up (19.1% vs. 34.4%, *p* = 0.044). There were also fewer patients with ASA > 2 in the revision group (29.6% vs. 55.1%, *p* = 0.017). In the revision group, there were fewer patients with NPH and a greater proportion of patients with Chiari I malformation or unspecific hydrocephalus compared with the non-revised group.

**Table 1:** Patient demographics

	All patients		Non-revision group		Revision group		*p* value	OR	95% CI
	*n* = 227	%	*n* = 180	%	*n* = 47	%			
Age: mean, years (± std)	58.4 (15.6)		59.8 (14.9)		53.0 (16.9)		*0.014*^*1*^		1.4, 12.3
Gender: male	104	45.8	87	48.3	17	36.1	0.136	1.7	0.9, 3.2
Status (end of follow-up): dead	71	31.3	62	34.4	9	19.1	*0.044*	0.45	0.2, 1.0
Smoker: yes	71^2^	37.0	53^3^	35.6	18^4^	41.9	0.452	1.3	0.7, 2.6
Comorbidities									
Hypertension	74	32.6	63	35.0	11	23.4	0.131	0.57	0.3, 1.2
Diabetes	23	10.1	20	11.1	3	6.4	0.339	0.55	0.2, 1.9
COPD	26	11.5	22	12.2	4	8.5	0.477	0.67	0.2, 2.0
Cerebrovascular disease	22	9.7	16	8.9	6	12.8	0.424	1.5	0.6, 4.1
Cardiovascular disease	29	12.8	25	13.9	4	8.5	0.325	0.6	0.2, 1.7
Tumour or malignancy	34	15.0	29	16.1	5	10.6	0.349	0.62	0.2, 1.7
ASA > 2	73^5^	50.3	65^6^	55.1	8^7^	29.6	*0.017*	0.3	0.1, 0.9
Aetiology/indication									
SAH	81	35.7	64	35.6	17	36.2	0.938	1.0	0.5, 2.0
NPH	55	24.2	50	27.8	5	10.6	*0.015*	0.3	0.1, 0.8
Tumour or malignancy	43	18.9	36	20.0	7	14.9	0.426	0.7	0.3, 1.7
Unspecified HC	11	4.8	6	3.3	5	10.6	*0.038*	3.5	1.0, 11.9
ICH	7	3.1	7	3.9	0	0	0.170	0.97	0.9, 1.0
Traumatic	8	3.5	6	3.3	2	4.3	0.760	1.3	0.6, 6.6
Meningitis	3	1.3	2	1.1	1	2.1	0.587	1.9	0.2, 21.8
Chiari I	7	3.1	3	1.7	4	8.5	*0.016*	5.5	1.2, 25.4
Other	6	2.6	3	1.7	3	6.4	0.073	3.0	0.7, 13.9
Pseudotumor cerebri	6	2.6	3	1.7	3	6.4	0.073	4.0	0.8, 20.6

^1^Mann-Whitney *U* = 3249, *p* = 0.014

^2^*n* = 192

^3^*n* = 149

^4^*n* = 43

^5^*n* = 145

^6^*n* = 118

^7^*n* = 27

*OD*, odds ratio; *CI*, confidence interval; *COPD*, chronic obstructive pulmonary disease; *SAH*, subarachnoid haemorrhage; *NPH*, normal pressure hydrocephalus; *HC*, hydrocephalus; *ICH*, intracerebral haemorrhage. Age both groups: median 61, min 18, max 89, range 71

### Surgical characteristics

Surgical characteristics are presented in Table 2. Mean surgery duration was 61.0 min ± 30.5, with no significant differences detected between the two groups (*p* = 0.133). The vast majority of implanted shunts were ventriculoperitoneal shunts (97.3%) with a strata (Medtronic) valve (89.8%), and there were no significant differences in the two groups in terms of shunt type or valve.

**Table 2:** Shunt surgery characteristics

	No. of patients	
	*n* = 226^1^	%
Surgery duration, mean (std)^2^	61.0 (30.5)	
Type of shunt		
VP	220	97.3
VA	2	0.9
CP	4	1.8
Type of valve^2^		
Medtronic strata II	202	89.8
Codman Certas	24	10.6
Bactiseal catheter	26	11.3
AB prophylaxis^3^	224	98.7
EVD prior to shunt	103	45.4
Kakarla score^4^		
1	102	49.5
2	83	40.3
3	21	10.2

^1^*n* = 226; in one patient, the shunt implant was immediately removed and surgery aborted due to intraoperative bowel perforation

^2^Median 55.0, range 204 (min 21, max 204)

^3^Total *n* = 227, missing data *n* = 3

^4^Missing data, *n* = 20

*VP*, ventriculoperitoneal shunt; *VA*, ventriculoatrial shunt; *CP*, cystoperitoneal shunt; *AB*, antibiotics; *EVD*, external ventricular drain

The accuracy of the intraventricular drain placement was determined by reviewing postoperative imaging and categorised in accordance with the Kakarla scoring system; 89.8% were characterised as Kakarla 1 or 2 score.

We found no significant differences between groups in terms of the Kakarla scores (1, 2 or 3), the number of primary shunt surgeries with a duration of less than 45 min or patients having received prior EVD to shunt surgery between the two groups.

### Shunt revisions

The revision rates of surgery are presented in Table 3. A total of 47 patients (20.7%) underwent a revision surgery at some point during the follow-up period. Thirty-two patients (14.1%) required their first revision surgery within 1 year from index surgery. Twenty patients (8.8%) underwent revision surgery more than 1 year after index surgery, either as a first revision or subsequent revision (revision surgery falling between > 1 year from index surgery and through 2018).

**Table 3:** Shunt revisions

		No. of patients	
		*n* = 227 (%)	
Patients with shunt revisions		47 (20.7)^1^	
Patients with 1st revision within 1 year		32 (14.1)	
Patients with revision > 1 year^2^		20 (8.8)	
Revision cause	Primary surgery	Revisions	All procedures
	*n* = 227 (%)	*n* = 90 (%)	*n* = 317 (%)
Infection	13 (5.7)	19 (21.1)	5.9
Proximal	11 (4.8)	27 (30.0)	8.4
Misplacement	11 (4.8)	13 (14.4)	4.0
Exploration	3 (1.3)	4 (4.4)	1.2
Skin perforation	2 (0.9)	3 (3.3)	0.9
Valve dysfunction	2 (0.9)	2 (2.2)	0.6
Length	1 (0.4)	1 (1.1)	0.3
Shunt conversion	1 (0.4)	8 (8.9)	2.5
Shunt removal	3 (1.3)	5 (5.6)	1.9
Distal	0 (0)	6 (6.7)	1.9
New shunt or valve	0 (0)	2 (2.2)	0.6

^1^A total of 47 patients needed revision surgery at some point in the follow-up period. 32 of these patients required a shunt revision within the *first year* of index surgery. 20 patients needed revision surgery after 1 year, some of whom had already been previously revised within 1 year after index surgery. 13 of those 20 patients had their *first* revisions after 1 year (not included in the table)

^2^Refers to *first* revisions from 1 year and 1 day until the end of observation time

The total number of procedures performed in the study period (including primary shunt insertion and shunt revisions) was 317. A total of 90 of these procedures were shunt *revisions* (28.3%), distributed among 47 patients with median 1.0 ± 2.0 and range 1–12. Fourteen out of 227 patients underwent ≥ 2 revisions (6.2%). The most common cause for the *first* revision was infection, occurring in 5.7%, followed by proximal failure/occlusion and misplacement, both occurring in 4.8% each. The most common cause of *overall* revision surgery was due to proximal failure/occlusion, accounting for 30.0% of revisions. Infection was the second most common cause for overall revisions, accounting for 21.1%. Misplacement accounted for the third most common cause for overall revisions with 14.4%.

Mean time from index surgery until the first revision in the 47 patients who required revision was 54.7 weeks ± 80.0 (median 11.0, range 0–257). The mean follow-up for all patients was 231.7 weeks ± 155.9 (median 217.0, range 0–561). Seventy-one out of 227 patients (31.3%) died during the follow-up period.

**Fig. 1:**
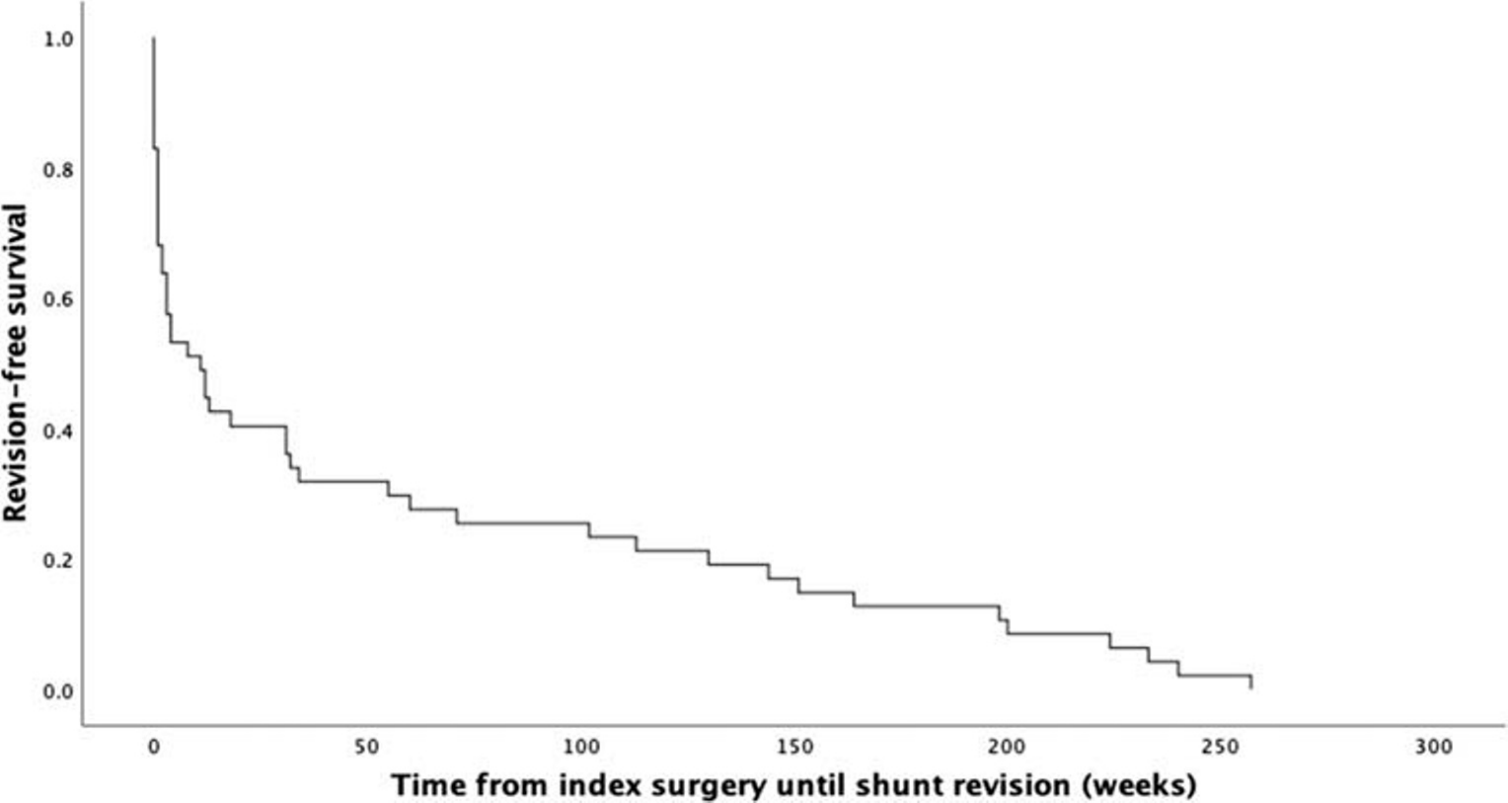
Revision-free survival in the revision group demonstrating time from index surgery until the first revision

Figure 1 demonstrates the revision-free survival probability (time from index surgery until first revision) with mean 54.7 weeks (95% CI 31.9, 77.6, median 11 weeks, 95% CI 1.3, 20.7). The initial steep slope indicates a high probability of revision occurring within the first year (52 weeks). Progressive flattening of the slope indicates the reduced probability for revision after 1 year, and no first revisions being performed after 250 weeks.

### Overall perioperative complications

An overview of perioperative complications classified with the Landriel Ibañez classification for all shunt procedures (combined primary shunt insertion and revision surgery) is provided in Table 4. A total of 103 patients (45.4%) experienced ≥ 1 complication(s). In total, 137 complications occurred (total 43.2%; medical 29.0%, surgical 14.2%) and were distributed among these 103 patients. Mild to moderate complications (grade I and II) were detected in 111 of the 317 procedures (35.0%). Severe or fatal complications (grade III and IV) were detected in 26 out of 317 (8.2%) procedures. UTI and pneumonia were the most common complications, occurring in 13.9% and 7.3% procedures, respectively.

**Table 4:** Postoperative complications

	Total	Medical	Surgical
	*n* = 317 (%)^1^	*n* = 92 (%)^1^	*n* = 45 (%)^1^
Grade I			
Ia	15 (4.7)	10 (3.2)	5 (1.6)
Ib	59 (18.6)	57 (18.0)	2 (0.6)
Grade II			
IIa	7 (2.2)	4 (1.3)	3 (0.9)
IIb	30 (10.2)	3 (0.9)	27 (8.5)
Grade III			
IIIa	10 (3.2)	4 (1.3)	6 (1.9)
IIIb	2 (0.6)	2 (0.6)	0 (0)
Grade IV	14 (4.4)	12 (3.8)^2^	2 (0.6)
No complications	180 (56.8)		

^1^Percent of total

^2^We registered all deaths occurring within 30 days, but only 2 deaths were directly related to a surgical complication. We have included the remaining 12 deaths within 30 days as medical complications

The Ia medical complications were exclusively cases of urinary retention. Surgical Ia complications consisted mainly of subdural effusion/subdural haematoma, managed conservatively, and two cases of new transient neurological deficits. UTI and pneumonia accounted for most of the Ib medical complications. Surgical Ib complications consisted of one wound infection requiring antibiotics only, and one patient with post-surgical seizures requiring anticonvulsants. The medical IIa complications were mainly patients with pleural effusions, requiring drainage. The surgical IIa complications consisted of one case of a dehiscent wound, requiring surgical closure under local anaesthesia, one patient requiring additional lumbar drainage and one case requiring temporary external drainage. Three cases of medical IIb complications were detected—pneumonia-causing atelectasis and requiring bronchoscopy in two patients and one patient suffering from an acute haemorrhage after undergoing tracheostomy, requiring intervention. The most common cause of surgical IIb complications was hardware misplacement (either proximally or distally), requiring reoperation. There were also five cases of track haematomas causing dysfunctional ventricular catheters, requiring revisions surgeries. Grade IIIa surgical complications consisted mainly of patients with CSF infection, verified by CSF specimens, requiring surgery with either externalisation or removal of the shunt. Four patients were re-intubated after surgery due to respiratory distress and respiratory failure and were classified as medical IIIa complications. Grade IV complications were classified as death occurring within 30 days of surgery regardless of cause. This was the case for fourteen patients; two patients died as a direct result of surgery/complication to surgery, and both died within a week of surgery (one instance of bowel perforation and one instance of acute aggressive meningitis). The remaining twelve patients died due to their underlying primary illness (acute subarachnoid haemorrhage, traumatic brain injury and primary extracranial malignancy) and one patient died of acquired illness/injury postoperatively. This latter case was a patient who received a shunt for NPH. The patient suffered a severe head trauma shortly after discharge, resulting in an acute subdural haematoma; due to severe clinical presentation, the patient was deemed not fit for surgical intervention and subsequently died within 30 days of shunt implantation.

Comparing index surgery procedures with revision surgery procedures, there was a higher occurrence of medical Ib complications in the index surgeries (*p* = 0.003). The rate of surgical IIb complications was significantly higher in the revision surgeries (*p* = 0.001).

A cox regression analysis failed to find a statistically significant association between the occurrence of shunt *revision* and underlying aetiology/indication, age or gender but found that cerebrovascular disease and hypertension increased the probability of shunt revision (*p* = 0.000 and *p* = 0.022 for cerebrovascular disease and hypertension, respectively). In a binary univariate analysis (complete case analysis, 145/227 patients included), age was inversely associated with the occurrence of revision *or* a grade III *or* IV complication (OR 0.972 for an additional year in age) but failed to show significance in a multivariate analysis. The cardiovascular and cerebrovascular disease were however found to increase the probability of revision surgery *or* a grade III *or* IV complication (*p* = 0.042, OR 6.1 and *p* = 0.04, OR 5.4 for cardiovascular and cerebrovascular disease, respectively), whilst other comorbidities, aetiology/indication and gender did not.

## Discussion

Despite apparent advancements in perioperative care and shunt technology, patients with hydrocephalus remain vulnerable with substantial susceptibility to shunt and surgery-related complications. In the present retrospective study, we found that approximately one-fifth of patients that underwent primary shunt surgery required revision surgery during follow-up. Proximal failure and infections were the most common causes of revisions. Although revision rates make up a significant portion of complications, we found that more than 40% of all shunt operations were followed by perioperative complications within 30 days. Thus, to reduce the risk of complications and subsequent revisions following shunt surgery, a multi-focused approach is necessary.

As experienced by others [12], most patients required revision surgery during the first year after index implantation. Some patients required revisions at a much later time, meaning if follow-up had been longer, the rate of shunt failure detected is likely to be higher. A review found that although there is a tendency of fewer shunt failures within the first years in adult patients, there is an increasing trend toward late failures [21]. This highlights the importance of including longer follow-ups in these types of studies. In the current study, only 127 of the 227 patients had more than 5 years of follow-up data available.

The current study demonstrates similar results in terms of revision rates to previous studies [4, 12], but generally lower rates than others where paediatric patients are included, even with subgroup analyses on adults [9, 18, 23]. Previous studies have indicated a young age as an independent risk factor for increased revision rates. We wished to avoid this as a possible confounding factor by not including paediatric patients. Additionally, the aetiology for HC in children is often different from adults, and therefore, only patients 18 and over were included, enabling us to study a more homogenous population. In our analysis, we *did* find that the patients in the group requiring shunt revision in general were about 6 years younger than patients in the group that did not require shunt revision. However, regression analysis failed to establish age as a significant independent risk factor. This might be due to the relatively small volume of patients in our study, especially in the revision group.

Shunt infection remains one of the most common and feared complications following shunt surgery and causes significant morbidity. Overall infection rates have commonly been reported to be around 5–6% in adult patients [12, 17, 19] but also significantly higher (8–15%) [13]. In a recent epidemiological study, the infection rate was reported to be around 12% [5]. In the current study, the infection was the most common cause for the *first* revision. Regression analysis did not reveal that factors such as age, gender or aetiology to be independent risk factors for the occurrence of revision surgery due to infection, unlike other previous studies [17]. Other reports have also indicated that subsequent shunt revisions increase the risk of further shunt infections in paediatric patients [3]. Proximal failure and misplacement (either proximally or distally) came close as the second most common cause for primary revision. In terms of revision rates, unlike our study, a recent Danish study interesting reported valve dysfunction to be the most common cause of complication/revision surgery but did not specify which valves were used [9].

The groups showed differences in the underlying aetiology (NPH, unspecified and Chiari), where NPH tended to have fewer incidences of revision surgery, whereas patients with Chiari or unspecified hydrocephalus (no obvious cause could be established) seemed more prone to shunt failure and revision surgery. However, our regression analysis failed to establish these aetiologies as independent risk factors for shunt revision. Other studies have shown that type of hydrocephalus or aetiologies is independent risk factors for shunt failure [9, 18, 23] with some suggest that NPH is a low-risk aetiology [18], whilst others have found the opposite [12].

The overall survival in the non-revision group was poorer than for patients in the revision group. This may be due to several factors; the non-revision group was generally older, and there was a higher rate of patients with ASA > 2, implying significant comorbidity in the non-revision group. This represents a possible source of selection bias; surgeons may have been more reluctant to perform revision surgery in this patient group. Additionally, shunt failure/complications may not have been properly recognised due to significant other comorbidities/concurrent illness that could explain or contribute to deterioration and leave patients deemed unsuitable for acute and/or revision surgery.

In previous studies, much attention is devoted to revision rates as the *sole* measure of shunt complications. In this study, we also aimed to review overall complications, also those that not necessarily resulted in revision surgeries. By careful review of patient records, we recorded all medical and surgical complications within 30 days postoperatively. We found that *overall* complication rates are much higher than revision rates. This is an important finding, suggesting that solely reviewing revisions rates in shunt surgery could undermine other important risk factors and downplay possible significant increased comorbidity postoperatively. Our overall complication rates and the occurrence of medical complications are similar to that of another study that recorded overall complication rates after elective brain surgery [22].

Instances of urinary retention, UTIs and pneumonia are common postoperative problems in these patients. Lack of data on whether patients were catheterised during surgery limits any assumption that urinary catheter use could be a problem; however, mean surgery duration was around 60 min, and therefore, one could argue that catheter use should not be used routinely in an attempt to reduce possible occurrence of urinary retention and UTI postoperatively. Similarly, focus on early mobilisation and lung physiotherapy could be an area of focus in an attempt to limit the occurrence of postoperative pneumonia. However, in this retrospective study, it is difficult to make any meaningful analysis on this; lack of attention to these complications and failure of systematic recording of such complications account for great uncertainty. More importantly, the great variety in the population included in this study, ranging from severely ill patients with long hospital stays in the ICU on ventilators to relatively healthy individuals with very short hospital stays will undoubtedly affect the occurrence of extracranial postoperative complications. Future studies could avoid this by performing separate analyses for elective shunt surgeries.

Furthermore, hardware misplacement accounts for a significant portion of surgical complications. Many have examined ways to optimise drain placement and perhaps routine utilisation of intraoperative ultrasound and/or neuronavigation could reduce the number of IIb complications [7, 11].

A review from 2000 described the advances of 50 years of shunt surgery at that time, suggesting overall shunt failure rates of 5% and infection rates to less than 1% as reasonable goals within the next decade [2]. Considering previously published data and the findings of this study, these figures appear to be over-ambitious and still far from reach despite being 20 years into this timeline. With knowledge of the complication rates and types of complications, we may be able to evaluate possible predictors of shunt failure. Furthermore, by not limiting complication rates to occurrences of revisions only, we might be able to move forward in an attempt to reduce complications in this patient group in the future. This can hopefully lead to overall better outcomes.

We did not review whether shunt complications and the burden of revision surgery affected comorbidity, cognitive function, occurrence or worsening of neurological deficits, ADL function, quality of life and work or educational status. One study examined some of these aspects (including quality of life, education and work participation) in hydrocephalic patients 40–45 years after shunt surgery, indicating great variance in these aspects [15]. Another smaller study examined adult NPH patients, showing great improvement in ADL and health-related quality of life [16]. In lieu of large-scale studies on adults, these would have been valuable aspects to assess.

This study has several limitations, one being the obvious retrospective design. To review complication rates in shunt surgery, it would have been preferred to follow the patients prospectively and to follow the entire patient population for a longer period of time to optimise means of comparison. Another limitation is the relatively small sample size that comes with the terrain of a medium-sized institution. Furthermore, one cannot exclude that surgeon’s choice in terms of decisions to perform primary shunt surgery and revision surgery and the possible bias this represents can impact on results; the degree of this is naturally difficult to determine in a retrospective design. We suspect some underreporting of the mild and moderate complications, particularly because we did not have access to information if patients were discharged from their local hospitals within 30 days directly to their homes or for instance to other institutions such as nursing homes. However, in terms of serious and fatal complications, these are more likely to be relatively accurately reported. One study reviewed the timeframes at which complications were detected and found that around 50% of serious or fatal complications were detected within 24 h [22]. Considering that shunt surgery requires patients to stay overnight in our department, the notion that serious complications would be reported seems to be a reasonable assumption. We understand that comparing such complication rates across centres can present challenges in a retrospective format. For instance, the indication for shunt exploration varies greatly among centres, often at surgeons’ discretion. Furthermore, especially mild complications will be susceptible to be missed due to differences in postoperative routines in different departments.

## Conclusions

Shunt revision remains a significant problem. Approximately 21% of patients needed revision surgery, most occurring within the first year after shunt insertion. Shunt infection, proximal failure and misplacement were the most common causes of revision surgery. Postoperative complications beyond revision rates in shunt surgery are rarely reviewed. A total of 45.4% experienced ≥ 1 complication(s), with extracranial infections such as UTI and pneumonia to be common findings postoperatively. Survival in the revision group was significantly longer than in the non-revision group, which may be due to different factors, such as older age and generally higher comorbidity score (ASA) in the non-revision group. Shunt complications should not be limited to merely shunt revisions. Additional increased knowledge about extracranial complications could contribute to a better understanding of the overall burden associated with shunt surgery. This would be valuable in terms of understanding overall patient outcomes and as a measure of successful or unsuccessful shunt surgery.
